# Ultrasonic Tethering to Enable Side-by-Side Following for Powered Wheelchairs

**DOI:** 10.3390/s19010109

**Published:** 2018-12-30

**Authors:** Theja Ram Pingali, Edward D. Lemaire, Natalie Baddour

**Affiliations:** 1 Department of Mechanical Engineering, University of Ottawa, Ottawa, ON K1N 6N5, Canada; tping033@uOttawa.ca (T.R.P.); nbaddour@uottawa.ca (N.B.); 2 Faculty of Medicine, University of Ottawa, Ottawa, ON K1H 8M5, Canada; 3 Ottawa Hospital Research Institute, Ottawa, ON K1H 8M2, Canada

**Keywords:** powered wheelchair, smart wheelchair, assistive technology, assistive navigation, human-following, social-following, ultrasonic triangulation, tethering

## Abstract

In social situations, people who use a powered wheelchair must divide their attention between navigating the chair and conversing with people. These conversations could lead to increased mental stress when navigating and distraction from maneuvering the chair. As a solution that maintains a good conversation distance between the wheelchair and the accompanying person (Social Following), a wheelchair control system was developed to provide automated side-by-side following by wirelessly connecting the wheelchair to the person. Two ultrasonic range sensors and three piezoelectric ultrasonic transducers were used to identify the accompanying person and determine their position and heading. Identification involved an ultrasonic beacon worn on the person’s side, at hip level, and receivers on the wheelchair. A drive control algorithm maintained a constant conversation distance along the person’s trajectory. A plug-and-play prototype was developed and connected to a Permobil F5 Corpus wheelchair with a modified Eightfold Technologies SmartChair Remote. Results demonstrated that the system can navigate a wheelchair based on the accompanying person’s trajectory, which is advantageous for users who require hands-free wheelchair control during social activities.

## 1. Introduction

Traditional joystick-controlled powered wheelchairs require users to understand their surroundings, perceive space (depth/color), and physically control the joystick [1]. Due to reduced vision, cognitive deficits, or motor-neuron diseases, a segment of users have difficulty using joysticks, which could lead to increased dependency on caregivers, mental stress, depression, social anxiety, and isolation [2,3]. Thirty-one percent of persons with mobility disorders are frequently depressed due to these factors [2]. Another issue is ‘distracted navigation’, which could lead to accidents such as tipping/falling and bumping into curbs, trees, or persons [4,5].

Recent advances in wheelchair technology have enabled smart wheelchairs (SW), an extension of powered wheelchairs that use an embedded computer and sensor systems to assist navigation [6]. This intelligent assistive device incorporates technology from autonomous mobile robots and requires minimum user involvement for navigation. A typical SW control has an input method, a processing device, and a drive controller. Input methods include joystick or gesture-based control using head posture, eye-gesture, voice commands [7,8,9,10]. SW assisted navigation can also include object following, also termed tethering. In this research, tethering is defined as the process of human-following to assist in powered wheelchair navigation.

Tethering techniques can be grouped into wired/contact tethering or contactless tethering. Contact tethering is loosely based on dog-on-a-leash, with a mechanical tether connecting the accompanying person (AP) to the wheelchair. For example, Chu et al. [11] used a mechanical string tether between a mobile robot and a person and measured the string tension and angle to calculate the person’s trajectory and determine the mobile robot’s direction and velocity. Na et al. [12] used a rod and reel mechanism to calculate a mobile robot’s speed for following. Contactless tethering would be advantageous over wired/contact tethering since wired/contact-based tethering could hinder free movement and maneuvering around obstacles between the AP and wheelchair. Contactless tethering use sensors like Lidar, cameras, or range-sensors to determine an object’s position and feed a control system to maneuver the wheelchair [11]. Examples include a human following mobile robot using laser range scanners to determine the shin position of a person in front, and then match the robot to the person’s trajectory [13] and a caregiver following wheelchair using omni-directional vision that wirelessly tethered the wheelchair to a caregiver moving beside the wheelchair [14].

Reviewing previous human-accompanying research [14,15,16,17,18], it can be concluded that tethering involves identifying the AP and determining the AP’s pose (position and orientation), which can be achieved using commercially available infrared range, ultrasonic range, cameras, or Lidar. For example, Kobayashi et al. [14] developed a caregiver following wheelchair that determines caregiver position using laser range sensors and extracted the contour of the shoulders as a means to track the caregiver movement, and an-omni-directional camera to identify the caregiver. The human following smart wheelchair [16] uses a laser range scanner (LRS) and an ultrasonic sensor ring to identify and determine the person’s pose respectfully. The ultrasonic sensor ring is used to determine the distance of the person in front of the wheelchair using triangulation. Ultrasonic sensors produce a high-frequency sound pulse to determine the distance of an object in its field-of-view. The distance is measured using Time-of-Flight (ToF) ([19], p. 139). Unlike light-based sensors (e.g., cameras, Lidar, infrared range sensors), ultrasonic sensors are not affected by the color or transparency/texture of the person’s clothes, can be used in low-light or no-light situations (e.g., in the dark or at night), are not affected by dust, smoke and mist [20], and have a resolution of 25 mm and a range from 152 mm to 6451 mm [21]. Lidar and LRS require a computer to process sensor information, making the system more expensive and bulkier.

This paper proposes a plug and play powered wheelchair control system to wirelessly tether the wheelchair to follow alongside an AP. This will allow the wheelchair user to converse with the AP without needing to physically control the wheelchair, thereby achieving the desired objective of social following, reducing mental stresses, and enhancing safety when maneuvering and navigating the wheelchair. This research implemented automated side-by-side following using ultrasonic tethering, with the SmartChair Remote [22] enabling seamless wheelchair control from the ultrasonic tethering (UT) system to the joystick, and vice versa. The main objective of this research was to develop a system that would initiate a casual proxemic communication distance [23] between the AP and the powered wheelchair, and test the ultrasonic tethering system for contactless tethering between a powered wheelchair and an AP.

## 2. Methodology

The design criteria for the social following powered wheelchair system were:

- Maintain conversation distance to minimum 60 cm and maximum 180 cm [23]
- Maintain tether when a person walks alongside the wheelchair and break tether when the person is no longer available
- Work with the powered wheelchair joystick controller, to allow user to retake joystick control at any time
- Perform as intended in low light
- Inexpensive and easy to connect and attach/detach to any powered wheelchair

**Figure 1:**
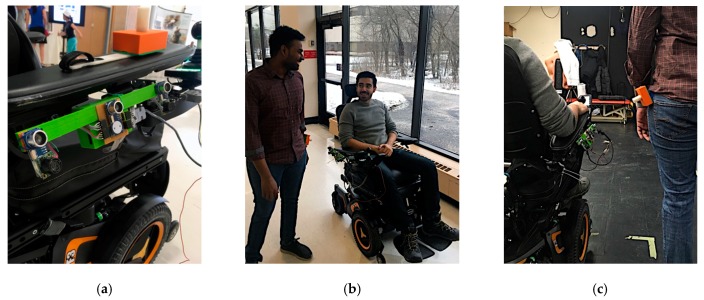
(**a**) Ultrasonic tethering system prototype mounted on a Permobil F5 corpus powered wheelchair, with AP beacon laying on the armrest, (**b**) Ultrasonic tethering system for social following, and (**c**) Ultrasonic tethering beacon placement on the accompanying person.

An ultrasonic tethering approach was selected to meet these design criteria. The prototype of the proposed system mounted on a Permobil powered wheelchair is shown in Figure 1.

### 2.1. System Architecture

The system architecture consisted of two main processes to achieve ultrasonic tethering: identify the AP and determine the AP’s pose (position and heading). Identification and pose estimation were achieved using commercially available ultrasonic sensors and a microcontroller. Ultrasonic sensors were chosen due to their small form-factor, ability to accurately detect objects within a short distance in different environments, and low processing power requirements for signal analysis.

#### 2.1.1. Identifying the Accompanying Person

**Figure 2:**
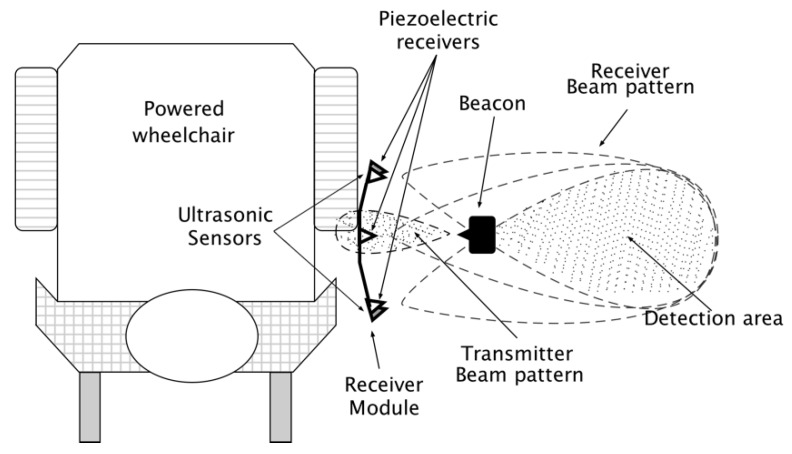
Detection area beside the powered wheelchair for AP identification. Beam patterns converge at the center of where the person is expected to walk.

**Figure 3:**
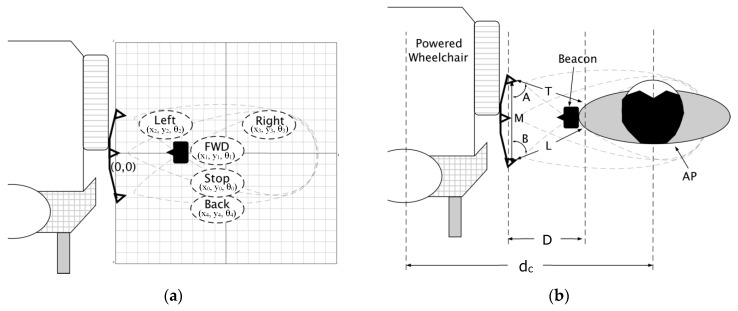
(**a**) AP position in coordinate space beside the wheelchair. x and y represent location coordinates and θ is the angle of a line from the origin to the point x, y. (**b**) Relationship between the AP conversation distance (d_c_) and tether distance (D).

In social situations, wheelchair navigation beside a person requires consistently identifying the person to tether and accompany, and avoiding wheelchair navigational error from intermittent tethering to by-standers or other objects beside the wheelchair. Three piezoelectric ultrasonic transducers on the wheelchair’s side and a beacon on the AP were used to identify the AP in the transducer field of view (detection area beside the wheelchair), as shown in Figure 2. AP beacon ultrasonic signals were received by piezoelectric ultrasonic receivers on the wheelchair’s side (Figure 3). The receivers produced an analog signal when the person was in the field of view.

#### 2.1.2. Determining Accompanying Person Position and Heading

Maintaining an appropriate conversation distance between the AP and wheelchair requires the relative AP position and heading. AP position beside the wheelchair was calculated by determining the distance between the wheelchair center and the AP, and calculating the AP angle with respect to the wheelchair. This position can be represented using a coordinate system with the receiver module as the origin (Figure 3a).

Position and heading are calculated using triangulation [24,25,26]. Two ultrasonic range sensors placed at the wheelchair side (facing the AP) were used to determine the distance from the sensors to the AP side (tether distance). The tether distance (D) is expressed as the total distance (conversation distance (d_c_)) subtracted from the sum of the distance from wheelchair’s sagittal plane to the receiver module (d_1_) and the distance from AP sagittal plane to beacon (d_2_). As illustrated in Figure 3b, a tether distance smaller than the conversation distance can be calculated using the Equation (1).

D = d_c_ − (d_1_ + d_2_) (1)

Ultrasonic range sensors produce an analog voltage proportional to the distance of an object closest to the sensor’s face. This distance was calculated from the sound pulse time-of-flight (ToF), by measuring the time for the pulse to travel from the sensor to the object and the time for the reflection from the object to the sensor [27]. The distance from the sensor to the person was calculated using Equation (2) [28]:

L, T = (Velocity of sound in air × ToF _(L, T)_)/2 (2)

### 2.2. Identification and Pose Detection Algorithm

**Figure 4:**
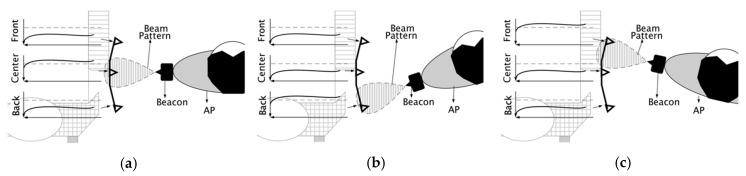
Ultrasonic transducer analog output while the person is (**a**) walking and stopping, (**b**) turning right, (**c**) turning left. Dashed line is high threshold.

AP identification and direction of motion were determined by comparing signals from the ultrasonic range sensors and piezoelectric ultrasonic transducers to thresholds. AP identification during walking, stopping, and turning occurred by comparing the piezoelectric ultrasonic receiver signal to a threshold (Figure 4). While walking, post-processed signals from the ultrasonic transducers are modulated as sine waves that are out of phase with each other, due to beacon angular motion at the hip (i.e., the beacon faces one transducer at a time, causing signal attenuation of the other transducers (Figure 4)).

**Figure 5:**
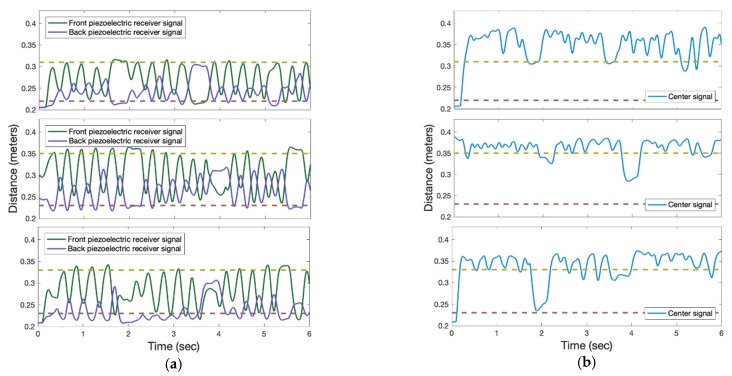
(**a**) Signals from front and back receivers for three trials. (**b**) Thresholding used on the center receiver signal to determine if the person is in-motion or static for three trials. Dashed lines represent the thresholds.

Figure 5 shows thresholds used by the receiver to identify the AP and determining if in-motion or static for three trials. The AP is identified when the center piezoelectric receiver signal is greater than the lower threshold. When walking straight, the center signal is greater than the high threshold. The AP stop and turning condition is when the center signal is between the low and high thresholds. The center signal amplitude is proportional to the angular motion at the hip.

**Figure 6:**
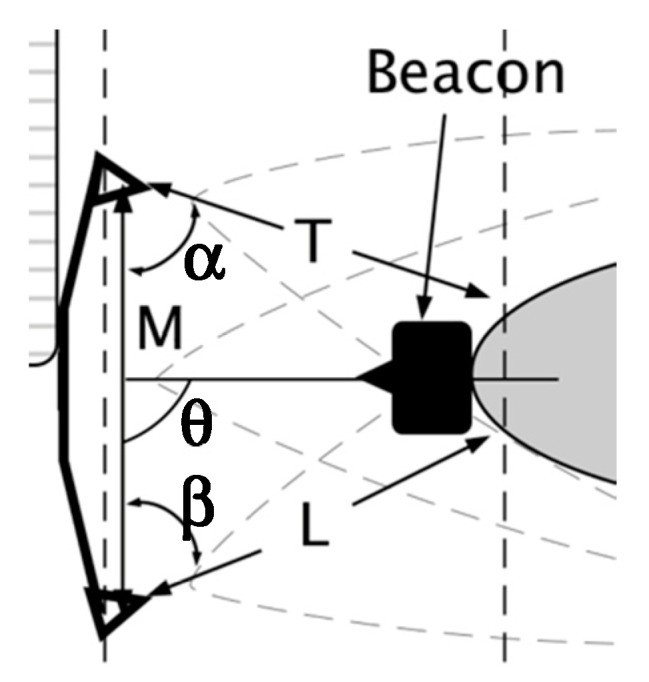
Nomenclature used for the algorithm. M is the distance between the front and back sensors. T and L are distances from the sensors to the AP side. α and β are angles formed between M and T or L and θ is the angle formed between M and the tether distance.

To determine AP position and heading, two ultrasonic range sensors were placed at a known distance (M) on the wheelchair’s side (Figure 6). Two distances to the AP (L, T) were determined from the sensor output. A line from the sensor center to the closest point on the AP was used to calculate two angles (α, β) using the Law of Cosines, Equations (3) and (4),

α = arccos ((T^2^ + M^2^ − L^2^)/2TM) degrees, (3)

β = arccos ((L^2^ + M^2^ − T^2^)/2LM) degrees, (4)

The angle of AP with respect to the center of the receiver module can be calculated using Equation (5):

θ = 180 − ((180 − (β + α)/2) + α),(5)

The distance between the closest part of the AP that reflects the ultrasonic sensor echoes and the center of the receiver module, can be calculated using Equation (6):

Tether distance = sqrt ((L^2^ + T^2^)/2M) (6)

## 3. Simulation

A simulation was designed to determine if ultrasonic tethering could be used for identification, position, and heading estimation. Using Matlab and Simulink, real world constraints such as noise and attenuation were incorporated to understand issues that could occur.

**Figure 7:**
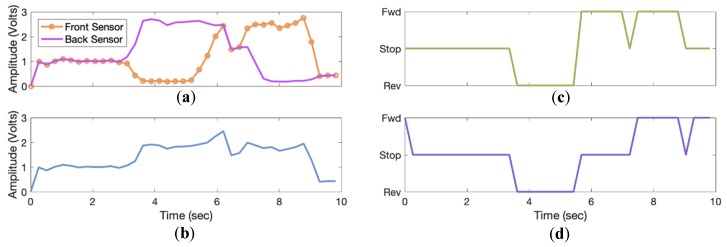
(**a**) Simulated post-processed front and back sensor signals, (**b**) calculated tether distance, (**c**) direction of left powered wheelchair motor driven using a differential drive system (directions correspond to tether distance and simulated sensor voltages), (**d**) direction of right powered wheelchair motor driven using a differential drive system (directions correspond to tether distance and simulated sensor voltages).

AP position and heading were modeled as signals from two range sensors. These analog signals were proportional to the AP distance beside the powered wheelchair and were based on Equation (2). If the person walks ahead, one of the sensors will output an analog voltage greater than the other (Figure 7a), which was later processed to determine the AP position and heading by calculating the tether distance based on Equation (6) (Figure 7b).

Signal amplification and filtering circuitry were simulated using Simulink-PSpice components. Amplification (gain) of 100 was implemented using an operational amplifier to amplify the input signal from millivolt range to volt range. The filtering circuit consisted of a diode-based envelope detector and an active low-pass filter (fcut = 50 Hz). The envelope detector was designed to remove all high-frequency noise and extract the signal envelope. Thresholding was used to determine the AP position and heading [29].

Simulation outputs were two motor voltage signals and an analog tether distance signal. The motor direction control signals consist of left and right motor signals (Figure 7c,d). Wheelchair direction based on the left and right motor signals are shown in Table 1.

## 4. Prototype

**Figure 8:**
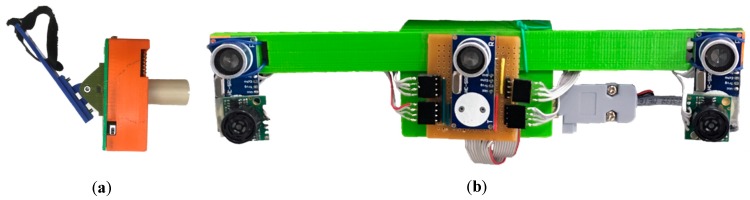
(**a**) Ultrasonic beacon for AP, (**b**) ultrasonic tethering receiver for powered wheelchair.

A prototype was developed using cost-effective 16 mm piezoelectric ultrasonic transducers, MaxBotix MB1010 ultrasonic range sensors, ATmega 328 microcontroller, and components such as operational amplifiers, resistors, capacitors, diodes, and 3-D printed cover (Figure 8).

### 4.1. Accompanying Person Beacon

The AP beacon transmits ultrasonic signal pulses toward the wheelchair (Figure 8a). This beacon hooks on the AP at the waist (belt, pocket, etc.), facing towards the wheelchair, and produces 40 kHz signal pulses using a piezoelectric ultrasonic transmitter and Atmel 328 based microcontroller as a pulse generator and voltage driver circuit. The microcontroller generates two 40 kHz pulses with 180-degree phase shift, using two built-in 16-bit timers. To achieve the required voltage to drive the 16 mm piezoelectric ultrasonic transducer, a voltage driver circuit based on a TTL to TIA/EIA232 converter was modified to generate 20 V (peak-to-peak) from a 5 V TTL logic. The beacon housing was 3-D printed using Polylactic Acid (PLA) and houses a 9 V battery.

### 4.2. Receiver on the Powered Wheelchair

**Figure 9:**
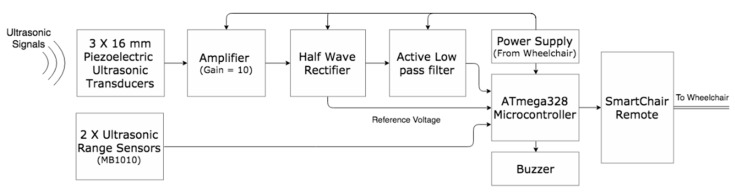
Receiver module hardware block diagram.

A receiver module with three piezoelectric ultrasonic receivers (spaced 12 cm apart), two MaxBotix ultrasonic range sensors (spaced 24 cm apart), signal amplification and filtering circuits, microcontroller, and 3-D printed box was fixed to the side of the wheelchair (Figure 8b). The MaxBotix range sensors were placed under the piezoelectric receivers. This module identified and detected AP position and heading in the ultrasonic transducer field of view. AP identification was achieved using three piezoelectric ultrasonic receivers placed in a concave pattern on the powered wheelchair’s AP side. AP position and heading were determined using two MaxBotix range sensors, oriented inward by 15-degrees. Range sensor placement was designed to point the sensors toward the center of a 30 cm radius circle, for optimal tether distance. These inexpensive ultrasonic sensors provided ranging from 0 to 254-inches, with 1-inch resolution with a narrow beam pattern [21]. All analog signals are converted to digital signals using the analog to digital converter inside the Atmel 328 based microcontroller. The microcontroller was also used to determine the wheelchair direction based on AP position. Figure 9 shows the receiver working block diagram.

### 4.3. Accompanying Person Feedback and Ultrasonic Tethering System Integration with the Powered Wheelchair

The receiver module was mounted, using 3D-printed U-clamps, on a Permobil F5 corpus powered wheelchair. A buzzer was included in the receiver module to produce a different tone for each change of mode. In situations where the tether is lost, the buzzer would sound an alarm for a few seconds, notifying the AP to move into the sensor field of view. In situations where deliberate loss of tether occurs, the buzzer alarm would be ignored, and the system would automatically switch to joystick control. Ultrasonic tethering system modes of operation are described in Table 2.

The onboard Atmel 328 based microcontroller transmits wheelchair direction commands as characters to a modified Eightfold Technologies SmartChair Remote [22] via serial communication. The system outputs characters based on the thresholding algorithm output: front (f), back (b), right (r), left (l), stop (s). The SmartChair Remote emulates a joystick and was connected in parallel to the wheelchair mechanical joystick, producing similar voltages as the joystick. SmartChair Remote modifications included a read data function from the ultrasonic tethering system.

### 4.4. Sensor Calibration

The ultrasonic tethering system calculates thresholds based on the ultrasonic receiver and ultrasonic range sensor information. For calibration, the AP stands next to the wheelchair, in the sensor field-of-view, for three seconds, at a comfortable distance for conversations between the AP and the wheelchair user. The system reads all sensor data and calculates low and high thresholds using Equation (7).

(7) $$\mathrm{threshold}=x\pm \left[\begin{array}{c} \frac{1}{n}\times \overset{n}{\underset{i=1}{\sum }}K_{d1}\times L_{i}+K_{d2}\times L_{i-1}\\ \frac{1}{n}\times \overset{n}{\underset{i=1}{\sum }}K_{d1}\times T_{i}+K_{d2}\times T_{i-1} \end{array}\right]$$

where, $K_{d1}$ and $K_{d2}$ are filter gains (0.8987 and 0.1013, respectively), L and T are the front and back sensor readings, n is the number of iterations, and x is the low or high gain.

## 5. Ultrasonic Tethering System Experimental Test Protocol

**Figure 10:**
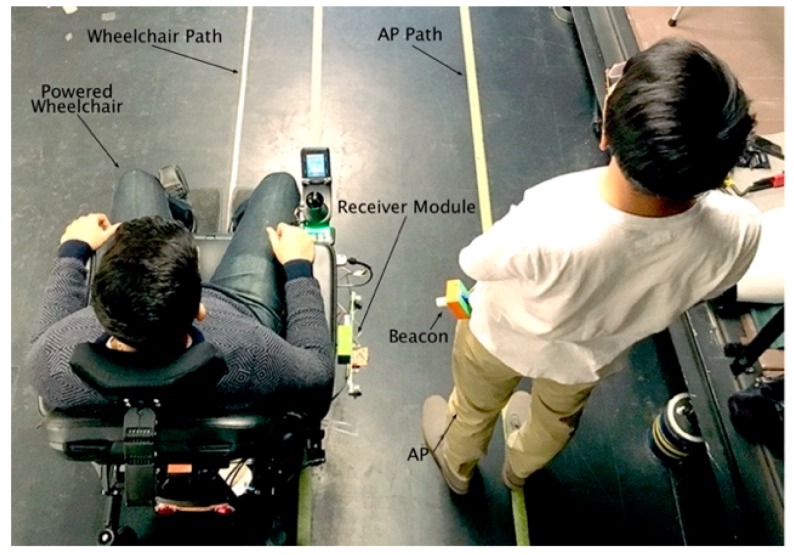
Ultrasonic tethering system performance test setup.

System performance was evaluated using sensor data acquired during three trials, where the AP and the wheelchair followed a fixed straight path for six seconds while data were acquired at 166 Hz. Two parallel lines were taped on the laboratory floor at 90 cm apart, the distance between the wheelchair center and person center (d_c_), as shown in Figure 10. The distance from wheelchair sagittal plane to the receiver module center (d_1_) was 40 cm and the distance from the AP sagittal plane to beacon (d_2_) was approximately 20 cm. The distance from the AP sagittal plane to beacon (d_2_) was measured using a tape measure, from the sagittal plane to the beacon’s face (in practice, this distance would be calculated by the system during calibration). From Equation (1), the tether distance was 30 cm. Wheelchair drive control was disabled to allow the user to drive the powered wheelchair along a straight path. Wheelchair direction commands were stored for observation and did not control the powered wheelchair. The AP walked beside the wheelchair, along the AP path at approximately 1 m/s [30]. Ultrasonic tethering system performance was evaluated using tether distance performance parameters (mean absolute error, average, standard deviation) and wheelchair direction command errors (total number of left and right turn commands that should be forward commands since the wheelchair was controlled by the user to move in a straight line).

## 6. Results

The tethering system prototype was constructed using circuits and hardware designed during the simulation. The mounting and beacon frames were 3D printed using PLA. The piezoelectric transducers produced a noisy signal that had an amplitude change of approximately 0.07 V for every decimeter; therefore, a circuit was developed with a non-inverting amplifier (op-amp with gain 10) an active low pass filter (fcut = 48.2 Hz with 3 dB attenuation), and demodulator using a diode based envelop detector. The signal required amplification with a gain of 10 to reduce clipping by the ATmega 328 based microcontroller’s 10-bit analog to digital converter.

**Figure 11:**
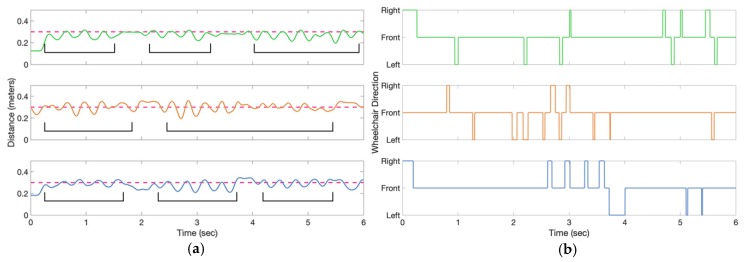
(**a**) Measured tether distance for the three walk trials (signals green, orange and blue for walk trial 1,2 and 3 respectfully). The dashed line represents the experimental tether distance (30 cm). The brackets indicate the effect of AP walking on the calculated tether distance, observed as sinusoidal waves. (**b**) Wheelchair direction commands for the three walk trials.

Figure 11a shows tether distances for three trials acquired while a person walked beside the wheelchair, while the user controlled the wheelchair. The tether distance had a mean absolute error of 6 cm with a standard deviation of 7.6 cm. Figure 11b shows the powered wheelchair direction commands for each trial acquired while the AP walked beside the wheelchair. The wheelchair direction commands were generated by a rule-based algorithm that compared tether distance and tether angles to thresholds (described in Section 4.4). The thresholds and wheelchair direction errors for the three trials are given in Table 3.

## 7. Discussion

Social following requires a powered wheelchair to follow beside a person at an appropriate conversational distance and location. This research successfully demonstrated that a prototype ultrasonic-tethering approach is viable for generating control signals to initiate and maintaining casual proxemic communication distance between the AP and powered wheelchair user.

A real time plug-and-play ultrasonic tethering system was designed with a modified Eightfold Technologies SmartChair Remote as the interface between the “ultrasonic tethering components and custom navigation software” and the wheelchair control system. The SmartChair Remote is a cross platform Bluetooth enabled joystick emulator that can be used to maneuver a powered wheelchair with a smart phone. The ultrasonic tethering system along with a SmartChair Remote can be connected to most commercially available powered wheelchairs, making the proposed solution broadly accessible to wheelchair users. The ultrasonic tethering system was developed with inexpensive components that were easily available in the market and all programming was implemented on an open source platform. The overall cost of developing the prototype was approximately $200 (CAD), which included the cost for manufacturing the 3-D printed parts (i.e., cases, clamps, holders), excluding the SmartChair Remote. Other contactless tethering techniques, such as using cameras and lidar sensors, require a computer with additional modules to convert digital signals to the required analog signals (and vice-versa) that are bulky and increase the overall system cost.

The ultrasonic system requires sensor calibration that stores and calculates the thresholds. These thresholds are based on a comfortable distance selected by the AP and the distance from the ground to the beacon. Sensor signals are affected by AP height, particularly the beacon height from ground level. To accommodate a range of AP heights, the prototype had hinges on the beacon and receiver modules to allow these components to rotate vertically and thereby provide a suitable sensor field-of-view. While this process would only take several seconds, the user may find the calibration phase and adjusting the beacon and receiver module angles as a hinderance.

The accompanying person’s arm natural swing while walking results in signal spikes or peaks due to the periodic obstruction of the beacon signal towards the receiver [31]. To address this, the front, center and back piezoelectric receiver signals were processed with a low pass filter to reduce signal spikes. This allows natural and comfortable human walking.

The walking trial results showed that tether distance varied due to the AP non-parallel motion (heading) with respect to the wheelchair. AP not walking in a perfect straight line, or the powered wheelchair moving off center to the pre-determined path, caused the measured average tether distance to be lower than the actual tether distance. Tether distance error was also caused by left to right sway due to person’s gait [32], where the range sensor detected the sway and output an oscillating signal that affected the calculated tether distance. This sinusoidal motion could cause an ultrasonic tethering system to change operating modes from available (tethered) to loss of tether or produce medial-lateral wheelchair undulations. In a social following control system, these deviations must be filtered, or removed by curve fitting and implementing a wheelchair trajectory planning algorithm [33,34], to avoid wheelchair lateral undulated motion when matching AP movement.

Safe and smooth wheelchair navigation is required for successful social following. Wheelchair kinematics may differ from the accompanying person’s motion since the person can abruptly start, stop, and turn while walking. However, powered wheelchairs require smooth starts and stops, and soft turns for the user’s safety and comfort. To achieve this, wheelchair trajectory planning and smoothing algorithms are needed for appropriate drive control [33,34].

## 8. Conclusions

Determining the accompanying person’s trajectory for social following poses a challenge to tethering-based wheelchair navigation. This research combined ultrasonic range sensors and active/passive components with a shared wheelchair-control algorithm to make a plug-and-play contactless tethering device. This was achieved using commercially available ultrasonic sensors, a microcontroller, 3-D printed components, and an Eightfold Technologies SmartChair Remote.

Principles of ultrasonic ranging were used to determine an accompanying person’s position and heading. A beacon was worn on the person’s lateral waist and ultrasonic sensors on the wheelchair determined the accompanying person’s pose by triangulation. Experimental results showed that the pose detection algorithm had a 6 cm tether distance error and the wheelchair direction error was 12%. The errors were due to pelvic sway during walking, and could be accommodated by modifying the thresholds and implementing wheelchair trajectory planning and smoothing algorithms. With these considerations, ultrasonic tethering can be a viable social following technology. Ultrasonic tethering can be advantageous for powered wheelchair users who require hands-free wheelchair control during social interactions.

## Figures and Tables

**Table 1:** Wheelchair direction of motion based on left and right motor signals produced by the simulation.

Left Motor Signal	Right Motor Signal	Wheelchair Direction
Stop	Stop	Stop
Forward	Forward	Forward
Forward	Reverse	Right
Reverse	Forward	Left
Reverse	Reverse	Backward

**Table 2:** Ultrasonic tethering system modes of operation.

AP Tethered	System Operation	Wheelchair Control/Motion	Buzzer Operation
Unavailable, not tethered	Not tethered	Joystick Control	No output
Available, Tethered	Tethered	Stop	1 s at 2 KHz
Available, Tethered	Tethered	Forward	No output
Available, Tethered	Tethered	Backward	No output
Available, Tethered	Tethered	Right	No output
Available, Tethered	Tethered	Left	No output
Loss of tether during operation	Not tethered	Stop + Joystick Control	2 s at 500 Hz

**Table 3:** The low and high thresholds and the wheelchair direction command errors produced by the drive control for each trial.

Trial	Low Threshold (m)	High Threshold (m)	% Error
1	0.22	0.31	12.0
2	0.23	0.35	9.8
3	0.23	0.33	14.1
